# Cadaveric Alkaloids

**Published:** 1881-01

**Authors:** 


### CADAVERIC ALKALOIDS.

The properties of the alkaline compounds which are formed during the decomposition of animal tissues have been the subject of an investigation by MM. Brouardel and Boutmy, which has been communicated to the French Association for the Advancement of Science (*Lancet*). Such substances have been called *ptomaines*, and they have been found in the bodies of individuals who have died a natural death, and also in those who have been poisoned. In cases in which the tissues are to be subjected to chemical analysis it is important to prevent the formation of these alkaloids, and the most efficient agent for this purpose is cold; and hence M. Brouardel has arranged for bodies which are to be subjected to analysis to be kept in the Morgue in chambers of refrigerated air. The "ptomaines" come into the general class of organic alkaloids, and many of them are most energetic poisons, others being quite innocuous. Though there are many distinct substances in the class, identical bodies are formed under very different conditions of putrefaction. The same alkaloid, for instance, was found in two individuals who were poisoned, the one by carbonic oxide, the other by prussic acid. A few are fixed, but the majority are volatile. A substance closely analogous to veratrine was found in a body which had been eighteen months in the Seine, and another in a goose which had been subjected to the heat necessary for cooking. Certain of these substances are clearly poisonous to man, and apparently cause the toxic effects which occasionally result from eating decomposing meat. Symptoms of serious poisoning occurred, for instance, in twelve persons who had partaken of a putrid goose which had contained a peculiar alkaloid, and one of these persons died in a few hours, after nausea and vomiting. These poisonous substances may be quickly formed, for in this case the goose had been purchased in the market in the morning of the day on which it was eaten.—*Louisville Med. News*, Oct. 23.

[The above may serve in explanation, to some extent, of the accidents as reported from time to time in which eating ice cream, chicken salad, etc., have resulted in distressing symptoms of poisoning. As various theories have been advanced upon the subject, it would be well to take into consideration the above theory, founded, it would seem, upon scientific principles. The copper vessel theory in which it is supposed the chemical formation of *verdigris* is the direct deleterious agent has never impressed itself very forcibly upon our minds, for the reason that the minuteness of quantity, and the fact that the poison is in a location which is undisturbed, as the vessel is rarely "scraped" to get out the "scorched dregs" precludes the idea of its acting very potently as a poison.—EDITOR.]

